# First-line afatinib vs gefitinib for patients with *EGFR* mutation-positive NSCLC (LUX-Lung 7): impact of afatinib dose adjustment and analysis of mode of initial progression for patients who continued treatment beyond progression

**DOI:** 10.1007/s00432-019-02862-x

**Published:** 2019-02-19

**Authors:** Martin Schuler, Eng-Huat Tan, Kenneth O’Byrne, Li Zhang, Michael Boyer, Tony Mok, Vera Hirsh, James Chih-Hsin Yang, Ki Hyeong Lee, Shun Lu, Yuankai Shi, Sang-We Kim, Janessa Laskin, Dong-Wan Kim, Catherine Dubos Arvis, Karl Kölbeck, Dan Massey, Angela Märten, Luis Paz-Ares, Keunchil Park

**Affiliations:** 1 West German Cancer Center, University Hospital Essen, University Duisburg-Essen, Hufelandstrasse 55, 45147 Essen, Germany; 2 Department of Medical Oncology, National Cancer Centre, 11 Hospital Drive, Singapore, Singapore; 3 Cancer Services, Princess Alexandra Hospital and Queensland University of Technology, 199 Ipswich Rd, Woolloongabba, Brisbane, 4102 Australia; 4 Department of Medical Oncology, Cancer Center of Sun Yat-Sen University, 651 Dong Feng Road, Guangzhou, 510060 China; 5 Department of Oncology, Chris O’Brien Lifehouse, 119-143 Missenden Road, Camperdown, NSW 2050 Australia; 6 Department of Clinical Oncology, The Chinese University of Hong Kong, Chung Chi Road, Hong Kong, China; 7 Department of Oncology, McGill University Health Centre, 1001 Décatie Blvd, Montreal, QC H4A 3JI Canada; 8 Department of Oncology, National Taiwan University Hospital and National Taiwan University Cancer Center, 7 Chung-Shan South Road, Taipei, 100 Taiwan; 9 Department of Internal Medicine, Chungbuk National University Hospital, Chungcheongbuk-do, Cheongju, South Korea; 10 Department of Lung Cancer, Shanghai Chest Hospital, Shanghai, China; 11 National Cancer Center/National Clinical Research Center for Cancer/Cancer Hospital, Chinese Academy of Medical Sciences and Peking Union Medical College, No. 17, Panjiayuan Nanli, Chaoyang District, Beijing, 100021 China; 12 Asan Medical Center, University of Ulsan College of Medicine, 93 Daehak-ro, Seoul, South Korea; 13 Department of Medical Oncology, BC Cancer Agency, 600 W 10th Avenue, Vancouver, BC V5Z 4E6 Canada; 14 Department of Internal Medicine, Seoul National University Hospital, 101 Daehak-ro, Jongno-gu, Seoul, South Korea; 15 Department of Oncology, Centre François Baclesse, 3 Avenue du Général Harris, 14000 Caen, France; 16 Department of Respiratory Medicine and Allergology, Karolinska University Hospital, 171 76 Stockholm, Sweden; 17 Statistics, Boehringer Ingelheim Ltd, Ellesfield Avenue, Bracknell, RG12 8YS UK; 18 Global Medicine, Boehringer Ingelheim International GmbH, Binger Straße 173, 55216 Ingelheim am Rhein, Germany; 19 Department of Medical Oncology, Hospital Universitario Doce de Octubre, CiberOnc, Universidad Complutense and CNIO, Av. Cordoba, s/n, 28041 Madrid, Spain; 20 Department of Medicine, Samsung Medical Center, Sungkyunkwan University School of Medicine, 50 Irwon- dong, Gangnam-gu, Seoul, 135-710 South Korea

**Keywords:** Afatinib, EGFR, NSCLC, Dose adjustment, Time-to-treatment failure

## Abstract

**Purpose:**

In the randomized phase IIb LUX-Lung 7 trial, afatinib significantly improved progression-free survival (PFS) and time-to-treatment failure vs gefitinib in patients with treatment-naïve epidermal growth factor receptor mutation-positive non-small cell lung cancer. We report post hoc analyses of tolerability-guided dose adjustment for afatinib and summarize the clinical characteristics of patients who continued afatinib/gefitinib beyond initial radiological progression in LUX-Lung 7.

**Methods:**

Patients received afatinib 40 mg/day or gefitinib 250 mg/day until investigator-assessed progression or beyond if beneficial. In case of selected treatment-related adverse events (TRAEs), the afatinib dose could be reduced by 10-mg decrements to minimum 20 mg (only dose interruptions were permitted with gefitinib).

**Results:**

All randomized patients were treated (afatinib, *n* = 160; gefitinib, *n* = 159). Sixty-three patients had afatinib dose reduction (< 40 mg/day; 47 within first 6 months). Dose reduction decreased TRAE incidence/severity (before vs after; all grade/grade 3: 100.0%/63.5% vs 90.5%/23.8%). There was no evidence of significant difference in PFS for patients who received < 40 mg/day vs ≥ 40 mg/day for the first 6 months [median: 12.8 vs 11.0 months; hazard ratio 1.34 (95% confidence interval 0.90–2.00)]. Twenty-four and 26 patients continued afatinib and gefitinib, respectively, beyond progression in target lesions; median time from nadir of target lesion diameters to initial progression was 6.7 months and 5.6 months. Of these patients, ~ 70% had objective response or non-complete response/non-progressive disease in non-target lesions at initial progression.

**Conclusions:**

Protocol-defined dose adjustment of afatinib may allow patients to remain on treatment longer, maximizing clinical benefit even in the presence of radiological progression.

**Electronic supplementary material:**

The online version of this article (10.1007/s00432-019-02862-x) contains supplementary material, which is available to authorized users.

## Introduction

Established first-line treatment options for patients with non-small cell lung cancer (NSCLC) and activating epidermal growth factor receptor (*EGFR*) mutations include: the first-generation reversible EGFR-targeting tyrosine kinase inhibitors (TKIs), gefitinib (European Medicines Agency 2018a; US Food and Drug Administration 2015a) and erlotinib (European Medicines Agency 2018b; US Food and Drug Administration 2010); the second-generation irreversible ErbB family blocker afatinib (European Medicines Agency 2018c; US Food and Drug Administration 2013) and the irreversible EGFR TKI dacomitinib (US Food and Drug Administration 2018); and the third-generation *EGFR* wild-type sparing, irreversible EGFR/T790M inhibitor, osimertinib (European Medicines Agency 2018d; US Food and Drug Administration 2015b). Until recently, there was a lack of prospective head-to-head comparisons of these agents.

The randomized phase IIb LUX-Lung 7 trial is, to the best of our knowledge, the first study to compare the irreversible ErbB family blocker (second-generation EGFR-targeting agent) with a reversible, first-generation EGFR TKI: in this case, afatinib was compared with gefitinib in treatment-naïve patients with advanced NSCLC harboring a common *EGFR* mutation (exon 19 deletion/L858R) (Park et al. 2016). The primary analysis of LUX-Lung 7 demonstrated that afatinib significantly improved the co-primary end points of progression-free survival [PFS; median 11.0 vs 10.9 months, hazard ratio (HR) = 0.73, 95% confidence interval (CI) 0.57–0.95; *P* = 0.017] and time-to-treatment failure (TTF; defined as the time from randomization to the time of treatment discontinuation for any reason; median 13.7 vs 11.5 months, HR = 0.73, 95% CI 0.58–0.92; *P* = 0.007) vs gefitinib (data cutoff: August 21, 2015) (Park et al. 2016). Analysis of overall survival (OS) demonstrated no significant difference in OS between the treatment groups [median 27.9 vs 24.5 months, HR = 0.86, 95% CI 0.66–1.12; *P* = 0.258 on April 08, 2016 (Paz-Ares et al. 2017) and HR = 0.85, 95% CI 0.66–1.09; *P* = 0.195 on December 05, 2016] (Corral et al. 2017).

The adverse event (AE) profiles for both afatinib and gefitinib in LUX-Lung 7 were consistent with previous experience, with no unexpected safety findings. As expected, diarrhea (afatinib vs gefitinib, all grades: 90.0% vs 61.0%; grade ≥ 3: 12.5% vs 1.3%) and rash/acne (all grades: 88.8% vs 81.1%; grade ≥ 3: 9.4% vs 3.1%) were more frequent with afatinib than gefitinib (Park et al. 2016), which is also consistent with observations with another irreversible second-generation EGFR TKI, dacomitinib (Wu et al. 2017). Increased alanine transaminase/aspartate transaminase (all grades: 24.5% vs 10.0%; grade ≥ 3: 8.8% vs 0%) was more frequent with gefitinib than afatinib (Park et al. 2016).

While the primary results of LUX-Lung 7 favored afatinib over gefitinib in a clinical trial setting, it is essential to consider factors that are likely to contribute toward treatment decisions in ‘real-world’ clinical practice. Regarding afatinib, a pertinent question is how should AEs, in particular diarrhea, be managed so that patients can remain on treatment for as long as they derive clinical benefit? Furthermore, is AE management with afatinib sufficiently effective in facilitating the ‘real-world’ clinical practice of continuing EGFR TKIs beyond radiological progression, in the absence of clinical deterioration? This is an important option for physicians and is recognized in current treatment guidelines (Novello et al. 2016), as it appears to reduce the risk of ‘disease flare’ (sudden increases in tumor growth and disease-related symptoms) in *EGFR* mutation-positive NSCLC patients with slow progressive disease (PD) (Chaft et al. 2011; Riely et al. 2007; Yap et al. 2017). In LUX-Lung 7, 35.0% of afatinib-treated and 29.6% of gefitinib-treated patients continued the assigned study treatment beyond radiological progression. For these patients, median duration of treatment beyond initial progression was 2.7 months (95% CI 1.9–4.3) and 2.0 months (95% CI 1.5–3.0), respectively (Park et al. 2016).

Previous studies have demonstrated that a well-established tolerability-guided afatinib dose adjustment protocol, which is facilitated by the availability of several dose strengths (European Medicines Agency 2018c; US Food and Drug Administration 2013), effectively mitigates afatinib-related AEs without impacting efficacy outcomes (Yang et al. 2016). Therefore, treatment discontinuation due to afatinib-related AEs is rare in clinical trials (6–8%) (Park et al. 2016; Sequist et al. 2013; Wu et al. 2014). Indeed, the effectiveness of tolerability-guided dose adjustment for AE management may also be reflected in the improvements in TTF observed with afatinib vs gefitinib in LUX-Lung 7 (Park et al. 2016).

In this sub-analysis of LUX-Lung 7, we further assessed the impact of tolerability-guided dose adjustment of afatinib with respect to AE management, patient-reported outcomes (PROs) and efficacy of treatment. We also evaluated the clinical characteristics of patients who continued afatinib or gefitinib treatment beyond initial radiological progression, to assess the potential for maximizing time on treatment for as long as patients derive clinical benefit.

## Patients and methods

### Study design and patients

Full details of the study design, treatments and assessments used in the LUX-Lung 7 trial have been published (Park et al. 2016). Briefly, LUX-Lung 7 (NCT01466660) was an international, multicenter, randomized, open-label phase IIb trial, conducted in 64 sites across 13 countries. Eligible patients were aged 18 years or older with: treatment-naïve pathologically confirmed stage IIIB/IV adenocarcinoma of the lung, a documented common activating *EGFR* mutation (exon 19 deletion/L858R), an Eastern Cooperative Oncology Group performance status of 0 or 1, at least one measurable lesion [Response Evaluation Criteria in Solid Tumors version 1.1 (RECIST v1.1)] and adequate organ function. The co-primary end points were PFS by independent central review, TTF and OS. Secondary end points included the proportion of patients with an objective response, tumor shrinkage and longitudinal change from baseline in health-related quality of life (QoL). The incidence and intensity of AEs, graded according to US National Cancer Institute Common Terminology Criteria for Adverse Events version 3.0 (NCI CTCAE v3.0), were also assessed.

LUX-Lung 7 was conducted in accordance with the provisions of the Declaration of Helsinki and Good Clinical Practice guidelines as defined by the International Conference on Harmonization. The study protocol was approved by an institutional review board or ethics committee at each participating center, and all patients provided written informed consent for participation in the trial.

### Treatment

Patients were randomized 1:1 to oral afatinib 40 mg/day or gefitinib 250 mg/day, stratified by *EGFR* mutation type (exon 19 deletion/L858R) and baseline brain metastases (present/absent). Afatinib dose escalation to 50 mg/day was permitted after 4 weeks of treatment in the absence of grade > 1 treatment-related AEs. In the event of the following treatment-related AEs, afatinib administration was paused for no more than 14 days until recovery to grade 1 or baseline, after which the afatinib dose was reduced by 10-mg decrements to a minimum dose of 20 mg: any grade ≥ 3 treatment-related AE, prolonged grade 2 diarrhea, grade 2 nausea or vomiting for ≥ 7 days despite supportive care or grade ≥ 2 worsening renal function (European Medicines Agency 2018c; US Food and Drug Administration 2013). Modifications in administration of gefitinib were permitted according to the summary of product characteristics, prescribing information or institutional guidelines. Gefitinib is only approved for administration in one dose formulation (European Medicines Agency 2018a; US Food and Drug Administration 2015a) and so no dose reduction schemes were implemented, but treatment interruptions were permitted. For each AE, the action taken with study treatment was recorded; however, this action taken only captured reductions or discontinuations and not treatment interruptions. In both treatment groups, the assigned study treatment could be continued beyond radiological progression (RECIST v1.1; by investigator assessment) in the case of continued clinical benefit as judged by the investigator. Following discontinuation of the assigned study treatment, crossover to the alternate study treatment was not permitted.

### Assessments and statistical analyses

All randomized patients [the intention-to-treat (ITT) population] from LUX-Lung 7 were included in the analyses. Three analysis time points were planned for LUX-Lung 7. The primary PFS/TTF analysis, planned after 250 PFS events (data cutoff: August 21, 2015), and the primary OS analysis, planned after approximately 213 OS events and a follow-up period of at least 32 months for patients still alive (data cutoff: April 08, 2016), have been published (Park et al. 2016; Paz-Ares et al. 2017). The final analysis will be undertaken at study completion (when all patients have completed treatment, or 5 years after the last patient was entered, whichever occurs first). Here, we present post hoc analyses using data from the primary PFS/TTF time point.

Analysis of the impact of afatinib dose adjustment at any time during treatment included assessment of: time to first dose reduction; exposure to afatinib 40, 30 and 20 mg; duration of treatment for patients whose dose was reduced to < 40 mg/day vs those whose dose was not reduced; and frequency and severity of the most common AEs pre- and post-dose reduction from 40 mg (according to the NCI CTCAE v3.0) (National Cancer Institute 2006). For patients whose dose was reduced to < 40 mg within, or remained on ≥ 40 mg/day for, the first 6 months of treatment (the time period when most dose reductions occur), post hoc analysis of baseline characteristics, PFS and PRO QoL outcomes [measured with the EuroQol 5 dimensions questionnaire (EQ-5D) and EuroQol Visual Analog Scale (EQ-VAS) scores] (Park et al. 2016) was conducted. PRO measures were performed until treatment discontinuation.

For patients who continued treatment beyond initial radiological progression (by investigator assessment), the baseline characteristics were summarized and compared with those of the ITT population; response to treatment in target and non-target lesions at the time of initial progression was also summarized. For those patients with initial PD in the target lesions, further summaries included: maximum percentage decrease in the sum of target lesion diameters before initial progression, best target lesion response, maximum percentage increase in the sum of target lesion diameters from nadir to initial PD, time to initial progression from the time point of the nadir and response of non-target lesions at the time of initial PD. The nadir of target lesion diameters was defined as the smallest sum on study of target lesion diameters (Eisenhauer et al. 2009).

Kaplan–Meier estimates and 95% CIs were calculated at planned imaging time points and used to estimate the median and quartile values (and 95% CIs) for PFS. A Cox proportional-hazards model was used to derive HRs and 95% CIs; groups were compared using a log-rank test. All other analyses were descriptive. Statistical analyses were performed with Statistical Analysis System (version 9.4).

## Results

### Patients and afatinib treatment exposure

In the LUX-Lung 7 trial, a total of 319 patients were randomized to afatinib (*n* = 160) or gefitinib (*n* = 159) and all patients were treated. Baseline characteristics for the overall population have been published and were similar between treatment groups (Park et al. 2016).

At the time of the analysis, 67 of 160 (41.9%) patients had at least one dose reduction of afatinib. Nine (5.6%) patients had afatinib dose escalations to 50 mg/day, but five (3.1%) of these patients experienced subsequent dose reduction to 40 mg/day, one of whom had a further reduction to 30 mg/day. In total, 63 (39.4%) patients had dose reductions to 30 mg, 21 (13.1%) of whom had further reductions to 20 mg. Twelve (19.0%) patients who had a dose reduction to < 40 mg/day and eight (8.2%) patients who received ≥ 40 mg/day throughout remained on treatment at the time of analysis. Median exposure to afatinib 40 mg was 7.4 months, to 30 mg was 7.6 months and to 20 mg was 7.6 months. Median time to first dose reduction was 1.9 months (range 0.4–28.6). The median duration of treatment for patients who received < 40 mg/day at any time was 16.3 months (range 1.1–39.4) and ≥ 40 mg/day throughout was 12.8 months (range < 0.1–40.1).

Of the 67 afatinib-treated patients who had dose reductions, 47 (70.1%) patients had a dose reduction to < 40 mg within the first 6 months of treatment. Baseline characteristics were generally similar between patients who had dose reduction to < 40 mg/day within, or remained on ≥ 40 mg/day for, the first 6 months of treatment (Table 1). Of the 47 patients who had a dose reduction to < 40 mg/day during this time, 36 (76.6%) were female and 30 (63.8%) were non-Asian. In the ITT population, 91 (56.9%) and 66 (41.3%) afatinib-treated patients were female and non-Asian, respectively (Park et al. 2016).

**Table 1:** Baseline demographics and disease characteristics of patients with/without afatinib dose reductions

Characteristic, *n* (%)	< 40 mg for the first 6 months (*n* = 47)	≥ 40 mg in the first 6 months (*n* = 113)
Gender		
Male	11 (23.4)	58 (51.3)
Female	36 (76.6)	55 (48.7)
Median age, years (range)	65 (37–86)	62 (30–83)
Race		
Asian	17 (36.2)	77 (68.1)
Black/African American	0 (0.0)	1 (0.9)
White	21 (44.7)	27 (23.9)
Missing^a^	9 (19.1)	8 (7.1)
Smoking status		
Never smoked	37 (78.7)	69 (61.1)
Ex-smoker	9 (19.1)	39 (34.5)
Current smoker	1 (2.1)	5 (4.4)
Baseline ECOG PS		
0	14 (29.8)	37 (32.7)
1	33 (70.2)	76 (67.3)
Histologic classification		
Adenocarcinoma	47 (100.0)	112 (99.1)
Mixed	0 (0.0)	1 (0.9)
Clinical stage at screening		
IIIB	0 (0.0)	8 (7.1)
IV	47 (100.0)	105 (92.9)
Metastases at screening		
Adrenal glands	5 (10.6)	7 (6.2)
Bone	25 (53.2)	55 (48.7)
Brain	6 (12.8)	20 (17.7)
Liver	7 (14.9)	9 (8.0)
Lung ipsilateral	26 (55.3)	60 (53.1)
Lung contralateral	17 (36.2)	48 (42.5)
Other	21 (44.7)	79 (69.9)

*ECOG PS* Eastern Cooperative Oncology Group performance status

^a^Patients recruited in French sites did not have their race recorded

### Impact of afatinib dose adjustment on treatment-related AEs, PFS and PROs

**Fig. 1:**
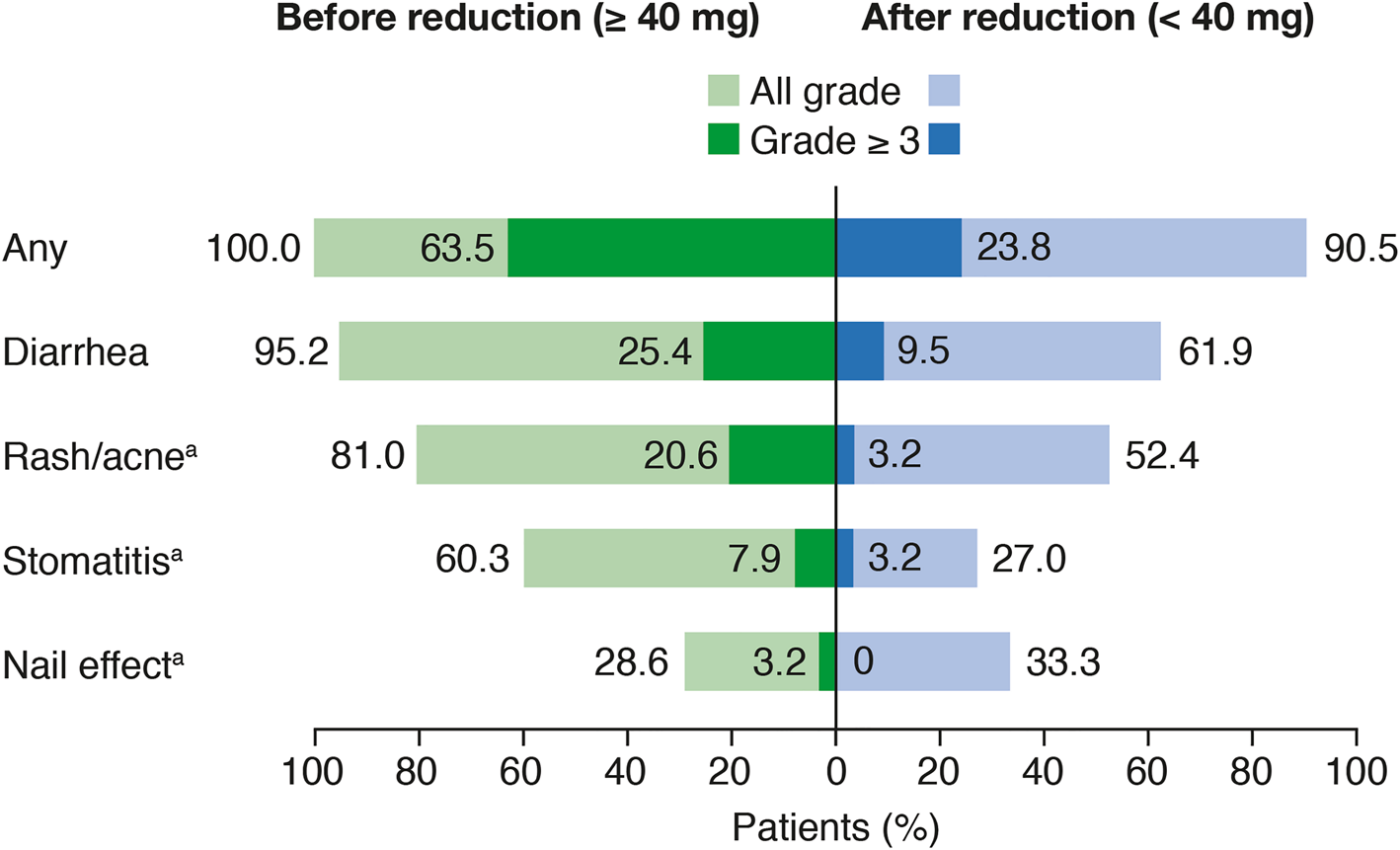
Key treatment-related adverse events (AEs) before and after afatinib dose reduction from 40 mg (*n* = 63). ^a^Grouped terms of AEs

As noted previously, if an AE led to discontinuation of study treatment, this was captured for both the afatinib and gefinitib treatment groups. If an AE led to dose reduction, this was also captured; however, this only applied to the afatinib treatment group as a dose-reduction scheme was only implemented for afatinib. Gefitinib label prescription only permitted treatment interruptions of up to 14 days if necessary for the management of certain AEs. Although all reported AEs were captured, it was not possible to identify which AEs led to a treatment interruption for either treatment group. All patients who received a dose reduction from the standard afatinib monotherapy dose of 40 mg/day (*n* = 63) experienced treatment-related AEs prior to dose reduction, 40 (63.5%) of whom experienced grade ≥ 3 treatment-related AEs (Supplementary Table 1). The most common treatment-related AEs among these patients were diarrhea, rash/acne, stomatitis and nail effect (grouped terms). Afatinib dose reduction to < 40 mg correlated with a decrease in the incidence of all grade and grade ≥ 3 treatment-related AEs overall [all grade: 90.5% (*n* = 57); grade ≥ 3: 23.8% (*n* = 15)] and across the key treatment-related AEs including diarrhea, rash/acne and stomatitis (Fig. 1; Supplementary Table 1).

**Fig. 2:**
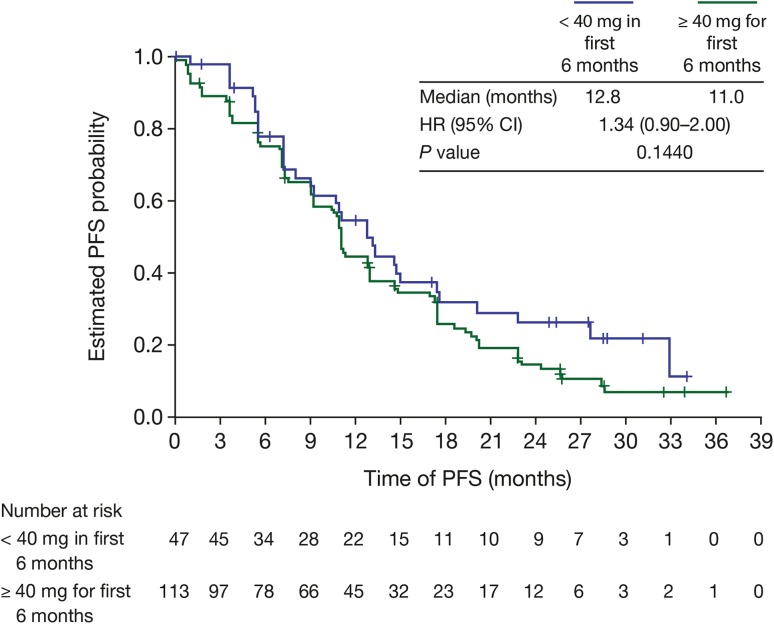
Median progression-free survival (PFS) in afatinib-treated patients who received a dose reduction to < 40 mg/day and in those who remained on ≥ 40 mg/day in the first 6 months of treatment

**Fig. 3:**
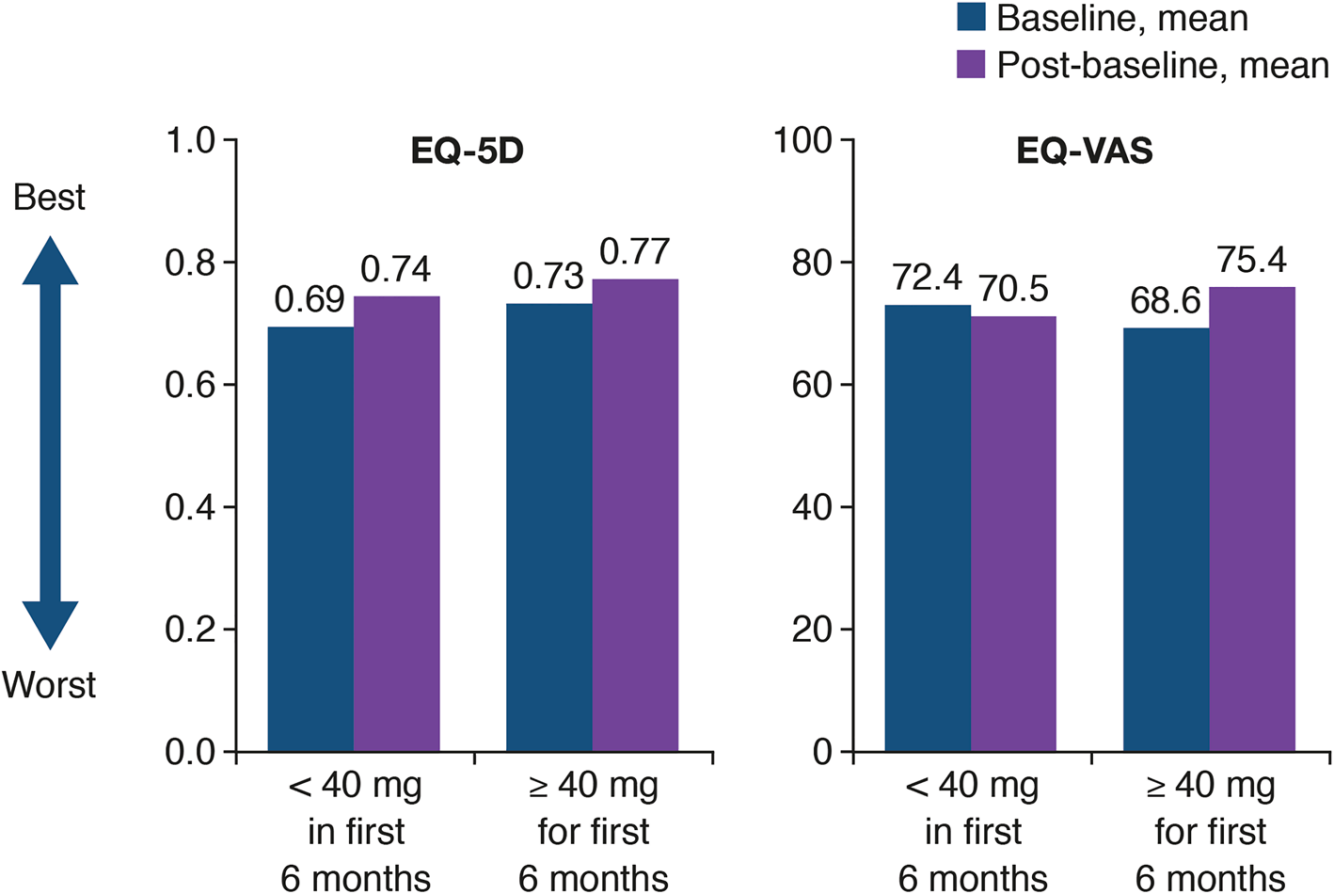
EuroQol 5 dimensions questionnaire (EQ-5D) and EuroQol Visual Analog Scale (EQ-VAS) scores in afatinib-treated patients who received a dose reduction to < 40 mg/day and in those who remained on ≥ 40 mg/day in the first 6 months of treatment

There was no evidence of a significant difference in PFS between patients who had a dose reduction to < 40 mg and those who remained on ≥ 40 mg afatinib for the first 6 months of treatment (12.8 vs 11.0 months, HR = 1.34, 95% CI 0.90–2.00; *P* = 0.144; Fig. 2). In addition, mean EQ-5D and EQ-VAS values at baseline and post-baseline were similar for patients who received < 40 mg/day in the first 6 months of treatment and for patients who received ≥ 40 mg/day for the first 6 months (baseline to post-baseline mean EQ-5D scores: < 40 mg, 0.69 to 0.74 and ≥ 40 mg 0.73 to 0.77; baseline to post-baseline mean EQ-VAS scores: < 40 mg, 72.4 to 70.5 and ≥ 40 mg, 68.6 and 75.4; Fig. 3).

### Treatment beyond progression

Baseline demographics and disease characteristics of both afatinib- (*n* = 56; 35.0%) and gefitinib-treated (*n* = 47; 29.6%) patients who continued their assigned study treatment beyond initial radiological progression were similar to those of the ITT population (Table 2). As previously reported in the primary PFS/TTF analysis (data cutoff: August 21, 2015), median PFS in the ITT population was 11.0 vs 10.9 months with afatinib vs gefitinib (HR = 0.73, 95% CI 0.57–0.95; *P* = 0.017) and median TTF was 13.7 months vs 11.5 months (HR = 0.73, 95% CI 0.58–0.92; *P* = 0.0073) (Park et al. 2016). In a subsequent analysis (data cutoff: April 08, 2016), similar improvements in PFS and TTF were observed in patients with exon 19 deletion and L858R-positive disease (Supplementary Fig. 1).

**Table 2:** Baseline demographics and disease characteristics of patients continuing treatment beyond initial PD and the ITT population

Characteristic, *n* (%)	Patients continuing treatment beyond initial PD		
	Afatinib (*n* = 56)	Gefitinib (*n* = 47)	ITT population (*n* = 319) (Park et al. 2016)
Gender			
Male	27 (48.2)	15 (31.9)	122 (38.2)
Female	29 (51.8)	32 (68.1)	197 (61.8)
Median age, years (range)	65 (39–86)	65 (38–86)	63 (30–89)
Race			
Asian	33 (58.9)	22 (46.8)	182 (57.1)
Black/African American	1 (1.8)	0 (0)	1 (0.3)
White	19 (33.9)	18 (38.3)	102 (32.0)
Missing^a^	3 (5.4)	7 (14.9)	34 (10.7)
Smoking status			
Never smoked	38 (67.9)	28 (59.6)	212 (66.5)
Ex-smoker	17 (30.4)	17 (36.2)	98 (30.7)
Currently smokes	1 (1.8)	2 (4.3)	9 (2.8)
Baseline ECOG PS			
0	20 (35.7)	12 (25.5)	98 (30.7)
1	36 (64.3)	35 (74.5)	221 (69.3)
Histologic classification			
Adenocarcinoma	56 (100.0)	47 (100.0)	317 (99.4)
Mixed	0 (0)	0 (0)	2 (0.6)
Clinical stage at screening			
IIIB	0 (0)	0 (0)	11 (3.4)
IV	56 (100.0)	47 (100.0)	308 (96.6)
Metastases at screening			
Adrenal glands	4 (7.1)	8 (17.0)	28 (8.8)
Bone	29 (51.8)	20 (42.6)	153 (48.0)
Brain	9 (16.1)	9 (19.1)	50 (15.7)
Liver	9 (16.1)	8 (17.0)	40 (12.5)
Lung ipsilateral	29 (51.8)	31 (66.0)	174 (54.5)
Lung contralateral	24 (42.9)	23 (48.9)	138 (43.3)
Other	31 (55.4)	25 (53.2)	204 (63.9)

*ECOG PS* Eastern Cooperative Oncology Group performance status, *ITT* intent to treat, *PD* progressive disease

^a^Patients recruited in French sites did not have their race recorded

A patient could have multiple reasons for radiological progression. Of those patients who continued the assigned treatment beyond initial PD, 24 (42.9%) afatinib-treated and 26 (55.3%) gefitinib-treated patients had documented PD in target lesions, 29 (51.8%) and 23 (48.9%) patients had a new documented lesion, and 20 (35.7%) and 16 (34.0%) had PD in non-target lesions at the time of initial PD (Supplementary Table 2). Twenty-four (42.9%) afatinib-treated and nine (19.1%) gefitinib-treated patients had a response [partial response (PR)/complete response (CR)] in target lesions at the time of initial PD, but had PD in non-target lesions and/or had new lesions (Supplementary Table 2).

**Fig. 4:**
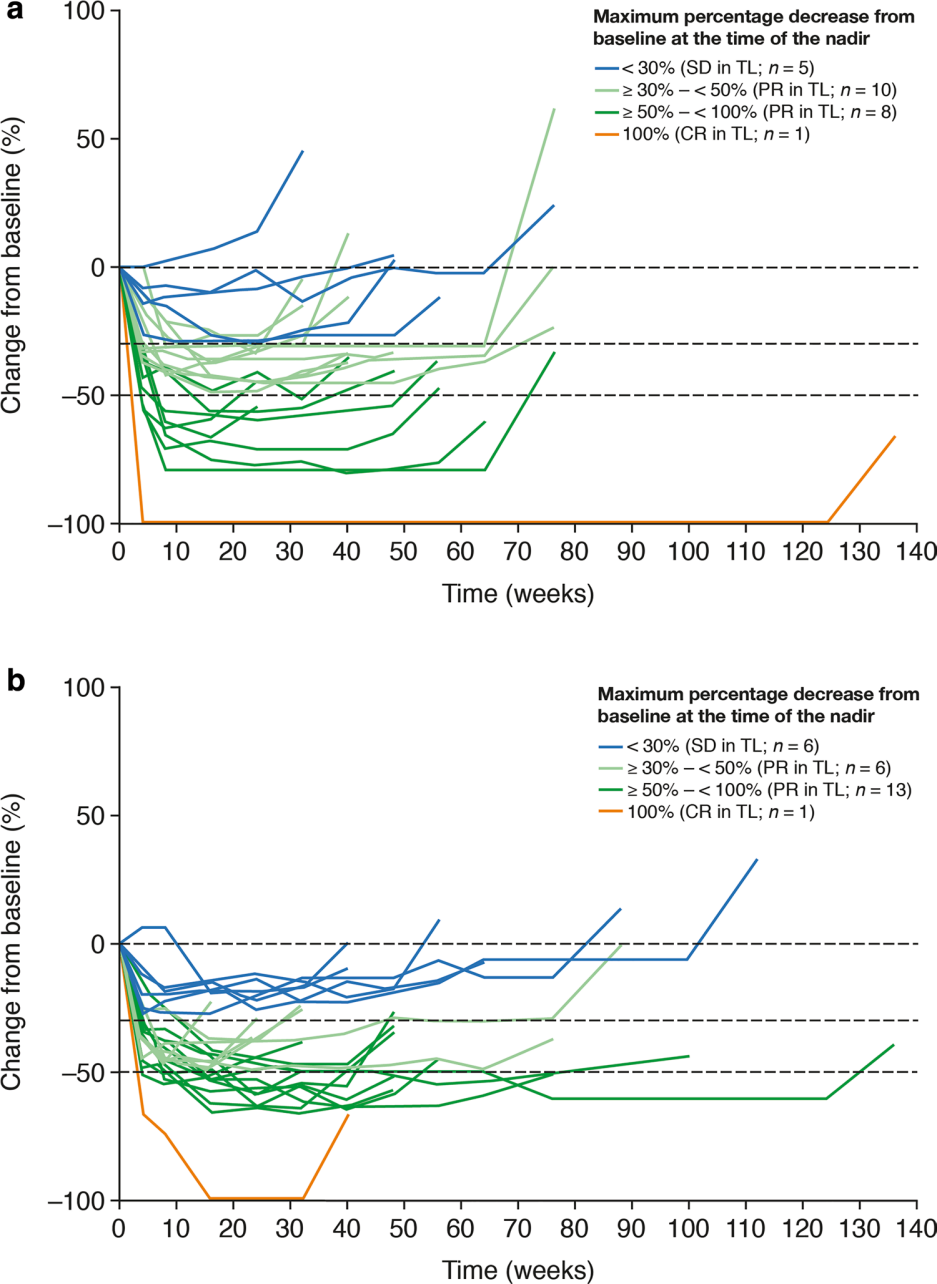
Percentage change from baseline^a^ in the sum of target lesion (TL) diameters for patients who continued afatinib (**a**) or gefitinib (**b**) beyond progressive disease (PD) in TLs. ^a^Until the point of initial disease progression

For the 24 afatinib-treated and 26 gefitinib-treated patients who had initial PD in target lesions and continued treatment beyond progression, the median maximum percentage decrease in the sum of target lesion diameters from baseline until the point of initial PD was 44.4% (range 0–100.0) with afatinib and 51.1% (range 19.4–100.0) with gefitinib (Supplementary Table 2). Of these, 19 patients (79.2%) in the afatinib arm and 20 patients (76.9%) in the gefitinib arm had an objective response (PR/CR) in target lesions at the time of the nadir (Fig. 4). Median maximum percentage increase in the sum of target lesion diameters from the nadir until the point of initial progression was 42.4% (range 20.0–220.0) with afatinib and 35.6% (range 20.4–100.0) with gefitinib. Median time from the nadir to initial PD in target lesions was 6.7 months (range 1.8–30.4) with afatinib and 5.6 months (range 1.8–16.5) with gefitinib (Fig. 4; Supplementary Table 2). There was a pattern toward either a pronounced response in target lesions at the nadir, or a long time to progression and/or a less pronounced increase in the sum of diameters of target lesions from the nadir to initial PD (Fig. 4). At the time of initial PD, two (8.3%) afatinib-treated patients with PD in target lesions demonstrated CR in non-target lesions, and 15 (62.5%) afatinib-treated and 18 (69.2%) gefitinib-treated patients with PD in target lesions demonstrated non-CR/non-PD in non-target lesions (Supplementary Table 2).

## Discussion

Effective management of AEs is an important aspect of the overall treatment strategy for patients with advanced NSCLC, with the goal of maximizing therapy exposure, and thus achieving optimal clinical benefit. Consistent with findings from other LUX-Lung studies (Yang et al. 2016), tolerability-guided dose reduction of afatinib in the LUX-Lung 7 trial led to decreases in the incidence and severity of key treatment-related AEs, particularly diarrhea and rash/acne.

The majority of afatinib dose reductions occurred within the first 6 months of treatment and were more common in females and non-Asian patients, even taking into account imbalances between gender and race in the ITT populations. In an analysis of afatinib dose reduction in the LUX-Lung 3 and 6 trials, afatinib plasma concentrations, which may vary among distinct subgroups of patients (e.g., based on gender) (Freiwald et al. 2014), were correlated with the incidence of dose reduction (Yang et al. 2016). Mean afatinib trough plasma concentration at the approved starting dose of 40 mg was higher in patients who had subsequent dose reduction, compared with patients who did not require dose reduction. Further, afatinib plasma concentrations for those who had dose reduction to 30 mg were similar to those who remained on the 40 mg dose (Yang et al. 2016). Although pharmacokinetic analyses were not conducted in the LUX-Lung 7 trial, the analyses from LUX-Lung 3 and 6, which consisted of a similar patient population and treatment setting, suggest that reducing the dose of afatinib in some patients may serve to mitigate excessive afatinib plasma exposure and thus reduce the burden of common treatment-related AEs. Given that gefitinib is only approved for administration in one dose formulation (European Medicines Agency 2018a; US Food and Drug Administration 2015a), no dose-reduction schemes were implemented for gefitinib in LUX-Lung 7, although dose interruptions were permitted for various reasons. It was not possible to identify AEs that led to a dose interruption due to an oversight in the design of the electronic case record form.

In LUX-Lung 7, there was no evidence of a statistically significant difference in median PFS with afatinib between patients who had a dose reduction to < 40 mg during the first 6 months of treatment vs those who remained on ≥ 40 mg during this time. Due to the inherent confounding in this analysis, as patients with early PD are unlikely to have been given the opportunity to reduce dose, some care should be taken in the interpretation of these results. There was no clinically meaningful difference in PROs for patients who received a dose reduction to < 40 mg during the first 6 months of treatment vs those who did not. Combined with the findings from the LUX-Lung 3 and 6 trials (Yang et al. 2016), these data indicate that dose reduction of afatinib is an effective strategy for the management of key treatment-related AEs, without negatively impacting the efficacy in patients with *EGFR* mutation-positive NSCLC. In the context of these data, it is important to note that there are currently no clinical data to support adaptation of the approved afatinib starting dose based on patient clinical characteristics, and underdosing at initiation of treatment may negatively affect the achievable clinical benefit with afatinib. Thus, the approved afatinib dose of 40 mg/day is recommended at treatment start and should only be modified based on individual patient tolerability. During the patient’s first few months of treatment, there should be frequent, vigilant follow-up and monitoring to ensure timely and appropriate dose adjustments.

Approximately, one-third of patients who were experiencing clinical benefit with afatinib or gefitinib continued their assigned treatments beyond investigator-assessed radiological progression (Park et al. 2016). The median duration of the assigned study treatment beyond PD in these patients was 2.7 months with afatinib and 2.0 months with gefitinib, which may suggest some benefit in continuing EGFR TKI treatment beyond radiological progression, with a numerically longer duration of treatment observed in the afatinib vs gefitinib treatment arm (Park et al. 2016). Around half of the patients who continued treatment beyond initial progression had no documented PD in target lesions, but instead had PD in non-target lesions and/or the occurrence of a new lesion, the latter of which might be controlled by localized therapies. At the time of initial progression, 42.9% of afatinib-treated and 19.1% of gefitinib-treated patients who continued treatment beyond progression had either a CR or PR in target lesions. Based on these data, from a clinical perspective it seems that progression in non-target lesions and/or the occurrence of a new lesion, especially in conjunction with responses in target lesions, may not impact a patient’s overall clinical disease control in a way that would justify a change of systemic therapy, with an uncertain efficacy of next-line treatment.

Those patients who continued afatinib or gefitinib treatment beyond initial radiological progression in target lesions demonstrated what might be viewed as a ‘slow progression’. In general, a gradual increase in the sum of diameters of target lesions over a median of 6–7 months between the time of the nadir and initial PD was observed. Around 70% of these patients demonstrated responses (CR/PR) or non-CR/non-PD in non-target lesions, which may suggest that progression in target lesions alone may not be the only factor requiring treatment discontinuation, because patients may still be able to derive clinical benefit from continued EGFR TKI treatment. Patients who continued their assigned treatment beyond progression in target lesions tended to demonstrate either a pronounced response in target lesions at the nadir, or a long time to progression and/or a less pronounced increase in the sum of target lesion diameters from the nadir to initial PD. Investigators deemed it possible for all patients who continued treatment beyond RECIST-defined progression in target lesions to derive further clinical benefit from continued treatment and may define these patients as ‘slow-progressors’, particularly those with a longer time to progression and/or less pronounced increase in target lesion size from the nadir to initial PD. The characteristics of patients who continued treatment beyond progression were similar in both the afatinib and gefitinib treatment arms, with regard to baseline characteristics and reason for PD (PD in target and/or non-target lesions, or occurrence of new lesions) (Park et al. 2016).

These findings from the LUX-Lung 7 study provide further support that tolerability-guided dose adjustment of afatinib reduces the incidence and severity of treatment-related AEs without affecting the efficacy or diminishing the effects of afatinib on PROs in patients with advanced *EGFR* mutation-positive NSCLC. Our further findings suggest that treatment beyond progression may allow patients who are deriving clinical benefit, for example, those with ‘slow progression’ or less clinically relevant new lesions in conjunction with a response in target lesions at the time of initial progression, to maximize time on EGFR TKI treatment. In conclusion, protocol-defined dose adjustment of afatinib may ultimately allow patients to remain on treatment longer, thus maximizing the clinical benefit, even in the presence of radiological disease progression.

## Electronic supplementary material

Below is the link to the electronic supplementary material.

Supplementary material 1 (DOCX 135 KB)

## Abbreviations

AE: Adverse events
CI: Confidence interval
CR: Complete response
ECOG PS: Eastern Cooperative Oncology Group performance status
EGFR: Epidermal growth factor receptor
EQ-5D: EuroQol 5 dimensions questionnaire
EQ-VAS: EuroQol Visual Analog Scale
HR: Hazard ratio
ITT: Intention to treat
NCI CTCAE: National Cancer Institute Common Terminology Criteria for Adverse Events
NSCLC: Non-small cell lung cancer
OS: Overall survival
PD: Progressive disease
PFS: Progression-free survival
PR: Partial response
PROs: Patient-reported outcomes
QoL: Quality of life
RECIST: Response Evaluation Criteria in Solid Tumors
SD: Stable disease
TL: Target lesion
TKIs: Tyrosine kinase inhibitors
TTF: Time-to-treatment failure
