# Supplementary material for: First-line afatinib vs gefitinib for patients with EGFR mutation-positive NSCLC (LUX-Lung 7): impact of afatinib dose adjustment and analysis of mode of initial progression for patients who continued treatment beyond progression
Source: J Cancer Res Clin Oncol. 2019 Feb 19;145(6):1569–79. doi: 10.1007/s00432-019-02862-x (PMC6527523; doi:10.1007/s00432-019-02862-x)
Supplement: Supplementary file 1 — Supplementary material 1 (DOCX 135 KB) [file 432_2019_2862_MOESM1_ESM.docx]

**SUPPLEMENTARY MATERIAL**

**Supplementary Fig. 1.** PFS^a^ (A and B) and TTF (C and D) in patients with common *EGFR* mutations (exon 19 deletion and L858R mutation)^b^


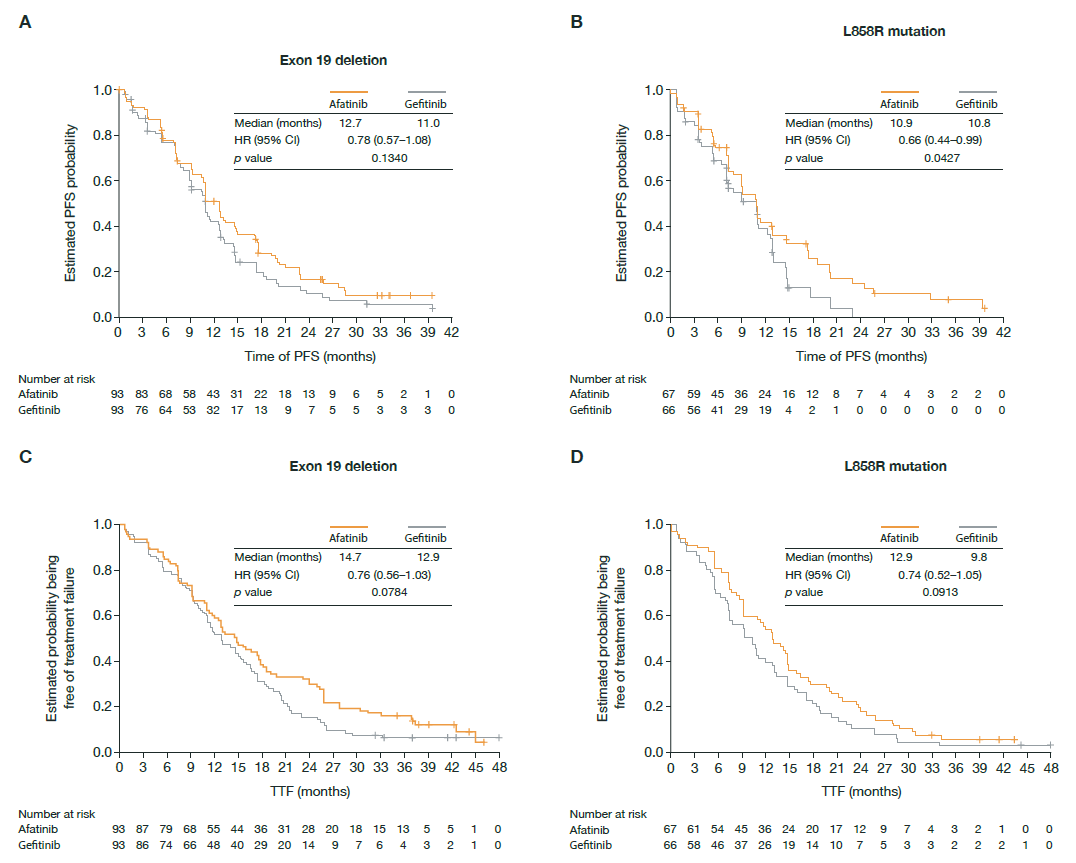


*PFS* progression-free survival; *TTF* time-to-treatment failure

^a^By independent review

^b^Data cutoff: April 08, 2017

**Supplementary** **Table 1** Treatment-related AEs pre- and post-afatinib dose reduction from 40 mg (> 5% incidence of all-grade AEs in either treatment arm)

| AE, *n* (%) | Onset prior to first dose reduction from 40 mg (*n* *=* 63) | | Onset after first dose reduction from 40 mg (*n* *=* 63) | |
| --- | --- | --- | --- | --- |
|  | All grades | Grade 3 | All grades | Grade 3 |
| Any | 63 (100.0) | 40 (63.5) | 57 (90.5) | 15 (23.8) |
| Diarrhea | 60 (95.2) | 16 (25.4)^a^ | 39 (61.9) | 6 (9.5) |
| Rash/acne^b^ | 51 (81.0) | 13 (20.6) | 33 (52.4) | 2 (3.2) |
| Stomatitis^c^ | 38 (60.3) | 5 (7.9) | 17 (27.0) | 2 (3.2) |
| Nail effect^d^ | 18 (28.6) | 2 (3.2) | 21 (33.3) | 0 (0.0) |
| Dry skin | 17 (27.0) | 0 (0.0) | 5 (7.9) | 0 (0.0) |
| Nausea | 15 (23.8) | 0 (0.0) | 8 (12.7) | 2 (3.2) |
| Pruritus | 15 (23.8) | 0 (0.0) | 8 (12.7) | 0 (0.0) |
| Fatigue^e^ | 12 (19.0) | 3 (4.8) | 13 (20.6) | 4 (6.3) |
| Vomiting | 11 (17.5) | 0 (0.0) | 4 (6.3) | 0 (0.0) |
| Decreased appetite | 9 (14.3) | 0 (0.0) | 3 (4.8) | 1 (1.6) |
| Epistaxis | 6 (9.5) | 0 (0.0) | 2 (3.2) | 0 (0.0) |
| Dry mouth | 4 (6.3) | 0 (0.0) | 1 (1.6) | 0 (0.0) |
| Dysgeusia | 4 (6.3) | 0 (0.0) | 1 (1.6) | 0 (0.0) |
| Dyspepsia | 4 (6.3) | 0 (0.0) | 1 (1.6) | 0 (0.0) |
| Alopecia | 3 (4.8) | 0 (0.0) | 6 ( 9.5) | 0 (0.0) |

*AEs* adverse events

^a^Including one (1.6%) grade 4 drug-related AE

^b^Grouped term including the following reported preferred terms: acne, blister, dermatitis acneiform, dermatitis bullous, drug eruption, eczema, erythema, exfoliative rash, folliculitis, rash, rash erythematous, rash follicular, rash macular, rash maculopapular, rash pruritic, rash pustular, skin disorder, skin erosion, skin exfoliation, skin fissures, skin lesion, skin reaction, skin toxicity and skin ulcer

^c^Grouped term including the following reported preferred terms: aphthous stomatitis, mucosal erosion, mucosal inflammation, mouth ulceration and stomatitis

^d^Grouped term including the following reported preferred terms: nail-bed infection, nail infection and paronychia

^e^Grouped term including the following reported preferred terms: asthenia, fatigue and lethargy

**Supplementary Table 2** Summary of response in target and non-target lesions and occurrence of new lesions at the time of initial PD for patients who continued to receive the assigned study treatment beyond initial radiological progression^a^

|  | All patients receiving treatment beyond PD | | Patients with PD in target lesions | |
| --- | --- | --- | --- | --- |
|  | Afatinib  (*n* = 56) | Gefitinib  (*n* = 47) | Afatinib  (*n* = 24) | Gefitinib  (*n* = 26) |
| Target lesion, *n* (%) |  |  |  |  |
| CR | 2 (3.6) | 2 (4.3) | - | - |
| PR | 22 (39.3) | 7 (14.9) | - | - |
| SD | 7 (12.5) | 6 (12.8) | - | - |
| PD | 24 (42.9) | 26 (55.3) | 24 (100.0) | 26 (100.0) |
| NE | 1 (1.8) | 6 (12.8) | - | - |
| Sum of diameters of target lesions, median (range) |  |  |  |  |
| Maximum percentage decrease from baseline |  |  | 44.4 (0–100.0) | 51.1 (19.4–100.0) |
| Maximum percentage increase from nadir until initial PD |  |  | 42.4 (20.0–220.0) | 35.6 (20.4–100.0) |
| Non-target lesion, *n* (%) |  |  |  |  |
| CR | 4 (7.1) | 2 (4.3) | 2 (8.3) | 0 |
| Non-CR/Non-PD | 30 (53.6) | 24 (51.1) | 15 (62.5) | 18 (69.2) |
| PD | 20 (35.7) | 16 (34.0) | 7 (29.2) | 7 (26.9) |
| NA/NE | 2 (3.6) | 5 (10.6) | 0 | 1 (3.8) |
| Occurrence of new lesion | 29 (51.8) | 23 (48.9) | 4 (16.7) | 7 (26.9) |

*CR* complete response; *NA* not applicable; *NE* not evaluable; *PD* progressive disease; *PR* partial response; *SD* stable disease

^a^By investigator assessment
